# Ribosomal Stress Couples with the Hypoxia Response in Dec1-Dependent Orthodontic Tooth Movement

**DOI:** 10.3390/ijms24010618

**Published:** 2022-12-29

**Authors:** Shigeru Nakamura, Keiji Tanimoto, Ujjal K. Bhawal

**Affiliations:** 1 Department of Public and Preventive Dentistry, Nihon University Graduate School of Dentistry at Matsudo, Chiba 271-8587, Japan; 2 Department of Translational Cancer Research, Research Institute for Radiation Biology and Medicine, Hiroshima University, Hiroshima 734-8553, Japan; 3 Department of Pharmacology, Saveetha Institute of Medical and Technical Sciences, Saveetha Dental College, Chennai 600077, India; 4 Department of Biochemistry and Molecular Biology, Nihon University School of Dentistry at Matsudo, Chiba 271-8587, Japan

**Keywords:** Dec1, ribosomal protein, HIF-1α, orthodontic tooth movement

## Abstract

This study characterized the effects of a deficiency of the hypoxia-responsive gene, differentiated embryonic chondrocyte gene 1 (Dec1), in attenuating the biological function of orthodontic tooth movement (OTM) and examined the roles of ribosomal proteins in the hypoxic environment during OTM. HIF-1α transgenic mice and control mice were used for hypoxic regulation of periodontal ligament (PDL) fibroblasts. *Dec1* knockout (*Dec1*KO) and wild-type (WT) littermate C57BL/6 mice were used as in vivo models of OTM. The unstimulated contralateral side served as a control. In vitro, human PDL fibroblasts were exposed to compression forces for 2, 4, 6, 24, and 48 h. HIF-1α transgenic mice had high expression levels of Dec1, HSP105, and ribosomal proteins compared to control mice. The WT OTM mice displayed increased Dec1 expression in the PDL fibroblasts. Micro-CT analysis showed slower OTM in *Dec1*KO mice compared to WT mice. Increased immunostaining of ribosomal proteins was observed in WT OTM mice compared to *Dec1*KO OTM mice. Under hypoxia, Dec1 knockdown caused a significant suppression of ribosomal protein expression in PDL fibroblasts. These results reveal that the hypoxic environment in OTM could have implications for the functions of Dec1 and ribosomal proteins to rejuvenate periodontal tissue homeostasis.

## 1. Introduction

The potential to produce new collagenous structural proteins, to differentiate into various types of cells, and to regulate the innate immune defense identifies periodontal ligament (PDL) fibroblasts as being of particular importance. PDL fibroblasts are mechanosensitive and account for the transduction of mechanical inputs into biological signals, resulting in remodeling of alveolar bone and the periodontium [1]. It is known that PDL fibroblasts can effectively and expeditiously acclimate mechanical forces in orthodontic tooth movement (OTM) and in alveolar bone homeostasis [2]. OTM-induced biological and biomechanical responses involve the regulation of transcription factors, cellular signaling pathways, morphogenetic circuitry, and inflammatory mediators of the immune system during periodontal tissue and alveolar bone remodeling [3,4].

In the early stages of OTM, the pro-inflammatory cytokine IL-1β intervenes in the process of periodontal tissue remodeling [5]. M1-like macrophages secrete iNOS and TNF-α in support of inflammation and alveolar bone resorption [6], thus ameliorating OTM. Pro-inflammatory macrophages might facilitate alveolar bone resorption, consequently influencing OTM [7].

The local microenvironment then undergoes temporary hypoxia because of a diminished blood supply, thus provoking remodeling [3,8]. Trabecular bone volume was decreased in HIF-1α deleted mice, since HIF-1α is involved in the activation of osteoclasts [9,10]. A hypoxic microenvironment is a necessary condition during the bone regeneration process [11]. HIF-1α directly induces the hypoxia-responsive gene differentiated embryo-chondrocyte 1 (Dec1/bHLHE40/Stra13/Sharp2), which is involved in regulating cell proliferation and apoptosis under hypoxia [12,13]. Numerous extracellular stimuli, including hypoxia, influence Dec1 expression [14].

The generation of physiological stress is also a common event of biomechanical forces in OTM [15]. Heat shock proteins (HSPs) constitute a conserved family of multiple cytoprotective proteins that respond to pathological and physiological stresses, such as heat shock, hypoxia, etc. [16]. Numerous HSPs have already been related to OTM [17,18]. Among them, the expression of HSP27 and its phosphorylation in the PDL of mice after orthodontic force application has been reported [18].

The identification of differentially expressed genes (DEGs) in PDL fibroblasts is crucial to enhance the understanding of the molecular mechanisms and biological functions involved in OTM. Herein, the single-cell data set GSE164157 of human teeth was downloaded from the Gene Expression Omnibus (GEO) database [19]. Gene ontology and KEGG enrichment and subsequently weighted co-expression network analysis (WGCNA) were used to identify hypoxia-responsive genes in PDL fibroblasts. Surprisingly, ribosomal proteins were the most enriched DEGs identified in those analyses. Ribosomes are mainly composed of ribosomal proteins, and expression patterns of those proteins are altered in mouse embryonic stem cells [20]. Ribosomal proteins are fundamental in the translation process, and anomalies in them can result in anemia [21]. Downregulation of RPL6 suppressed gastric cancer growth [22]. RPS7 plays a significant role in MDM2-p53 interactions [23,24] and in MAPK and PI3K/AKT signaling [25]. Environmental stressors can modulate gene expression to trigger ribosomal proteins to link with ribosomes. Ribosomal stress occurs as a direct consequence of ribosome biogenesis dysfunction [26,27].

In vitro cellular experiments with different mechanical stimulations have been extensively studied in OTM [28]. Currently, a variety of genetically engineered mice have also been used to study OTM [29,30]. Gene-targeted mouse models have significantly improved our understanding of the multicellular functions of OTM. Thus, the objectives of this study were: (i) to characterize the regulation of HSPs in PDL fibroblasts in a hypoxic environment during OTM, (ii) to investigate the essential role of ribosomal proteins in the hypoxic regulation of periodontal fibroblasts, (iii) to determine the effects of hypoxia-responsive genes and the expression of ribosomal proteins in HIF-1α transgenic mice, (iv) to illustrate the function of hypoxia-responsive genes in the genetic control of OTM, and (v) to elucidate the crosstalk among environmental stressors (hypoxia, heat shock, inflammation, ribosomal) that function in the orthodontic force environment.

## 2. Results

### 2.1. Analysis of Single-Cell Sequencing Data

**Figure 1:**
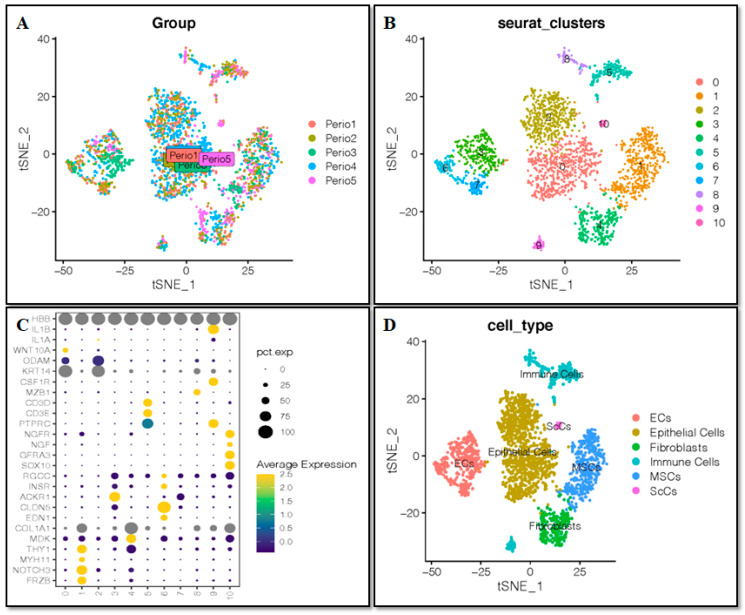
Single-cell analysis and cell annotation. (**A**) Sample-to-batch effect. (**B**) 11 clusters of cells were identified. (**C**) Marker gene expression in each cluster. (**D**) Cells are annotated into six cell types.

Different samples were integrated to analyze the single-cell sequencing dataset GSE164157 of human teeth. Subsequent analysis was based on the high-quality integration effects among 5 samples without an obvious batch effect (Figure 1A). The kNearest Neighbor (KNN) clustering algorithm was applied to cluster all cells into 11 distinct clusters (Figure 1B). In accordance with the surface marker genes of different cell types (Supplementary Table S1), their expression patterns were noted in the various clusters (Figure 1C) and definitively established 6 cell types—endothelial cells (ECs), epithelial cells, fibroblasts, immune cells, mesenchymal stem cells (MSCs), and Schwann cells (ScCs) (Figure 1D).

### 2.2. Hypoxia-Activated Phenotype of Different Cell Types in Periodontal Tissue

**Figure 2:**
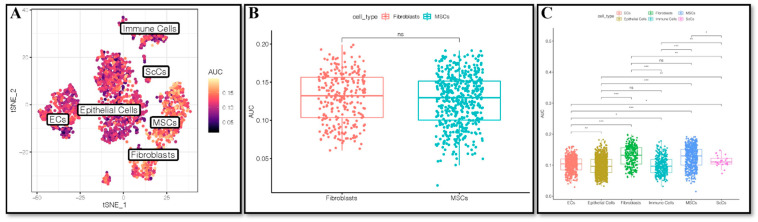
Activation of hypoxia in cell types. (**A**) Tsne diagram of the activation of hypoxia in cell types. (**B**) Activation of hypoxia in fibroblasts and MSCs. (**C**) Boxplots of the activation of hypoxia in cell types. * *p* < 0.05, ** *p* < 0.01, *** *p* < 0.001. ns, not significant.

As shown in Figure 2A–C, the hypoxia phenotype was mainly activated in fibroblasts and MSCs, and the median AUC value of hypoxia activation was the highest in fibroblasts. These results suggest that hypoxia is closely related to fibroblasts in periodontal tissue.

### 2.3. Weighted Gene Co-Expression Network Analysis (WGCNA)

**Figure 3:**
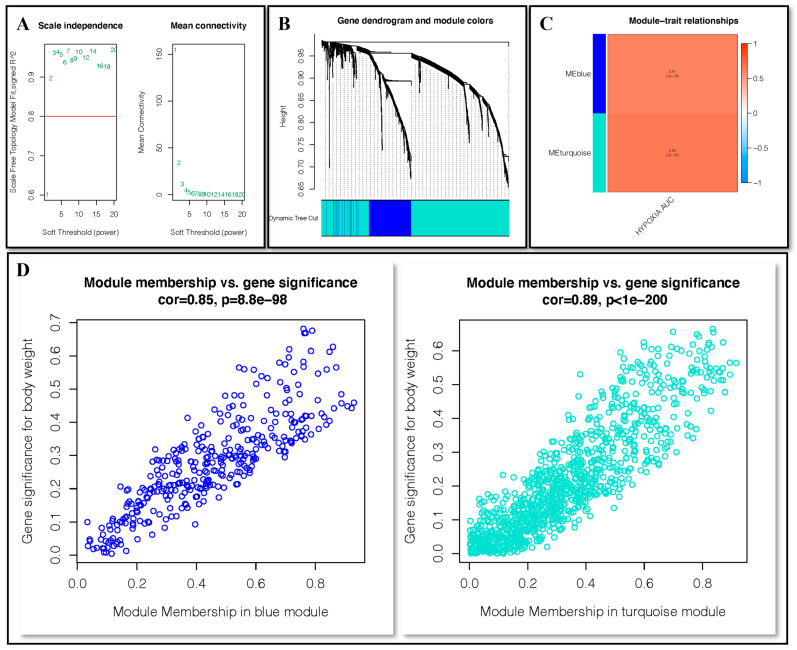
WGCNA analysis. (**A**) Setting the soft threshold. (**B**,**C**) Genes were clustered into two modules, blue and turquoise. (**D**) Correlation between module membership and gene significance in blue and turquoise modules.

WGCNA analysis of fibroblasts was employed to identify the gene modules closely related to hypoxia. With the minimum number of module genes set as 50, a total of 2 non-gray modules, deepSplit as 3, were given by setting the soft threshold as 2 (Figure 3A,B). MEblue and MEturquoise were intricately connected to the hypoxia score in the non-gray module (Figure 3C). The correlation between gene significance of body weight and membership in the blue module was 0.85 (*p* < 0.001) and in the turquoise module was 0.89 (*p* < 0.001) (Figure 3D).

### 2.4. KEGG and GO Enrichment Analysis

**Figure 4:**
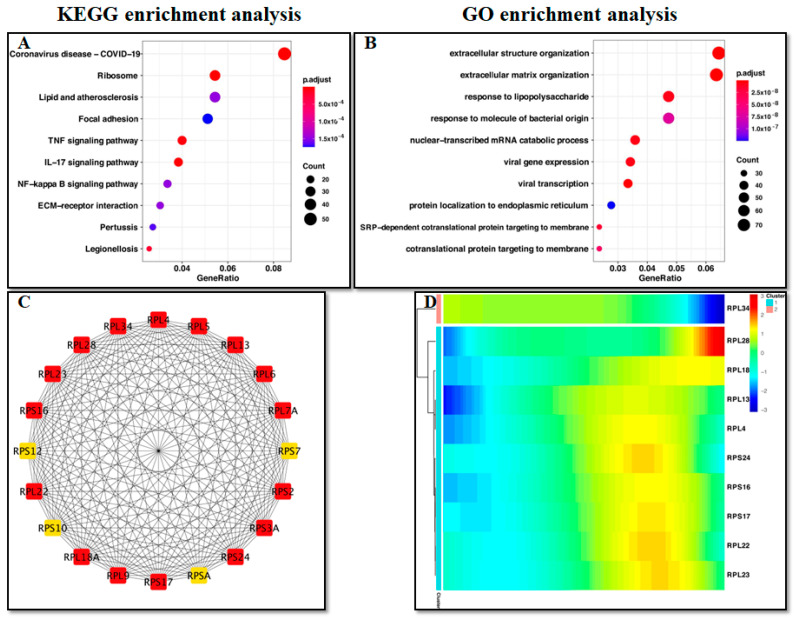
Exploring the functions of genes related to fibroblast differentiation. (**A**) KEGG enrichment analysis. (**B**) GO enrichment analysis. (**C**) PPI analysis of the top 20 DEGs. (**D**) Expression of the top 10 DEGs associated with fibroblastic differentiation.

The genes in the blue and turquoise modules were mainly related with ribosomes and extracellular organization (Figure 4A,B). Analysis of protein–protein interactions revealed the top 20 DEGs (Figure 4C). As fibroblasts differentiate, among the top 10 DEGs, RPL34 gene expression gradually decreased, while expression of the other 9 ribosomal proteins increased (Figure 4D).

### 2.5. HIF-1α Facilitates the Expression of Dec1 and HSP105 and Triggers Ribosomal Protein Expression in Mice

**Figure 5:**
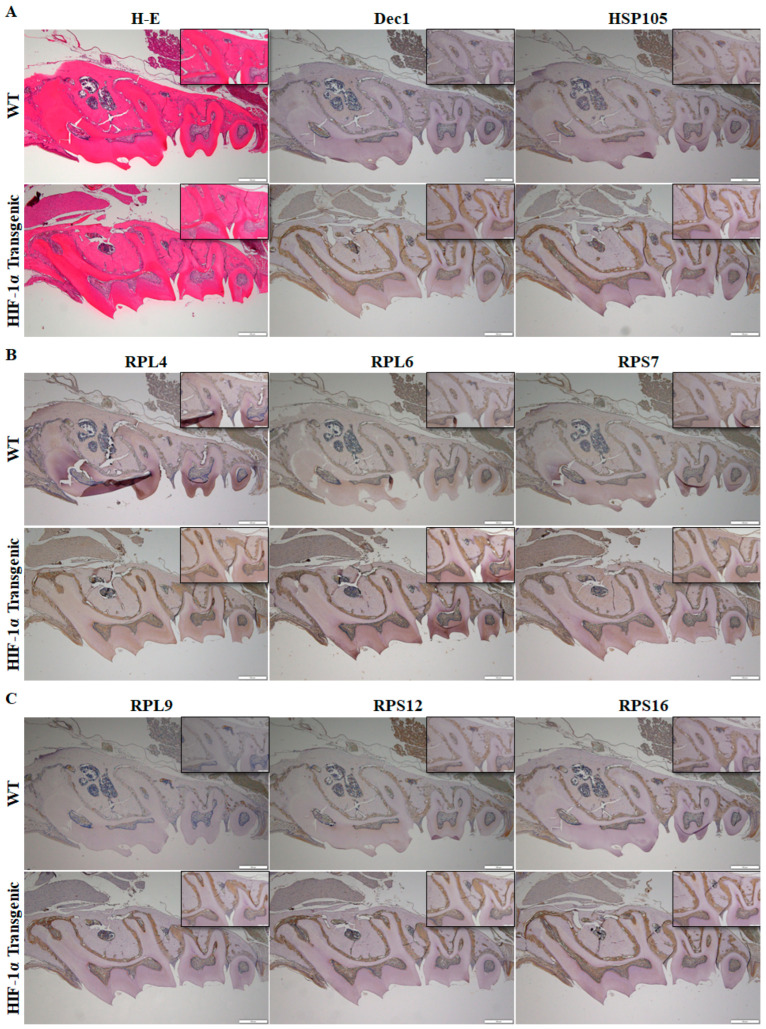

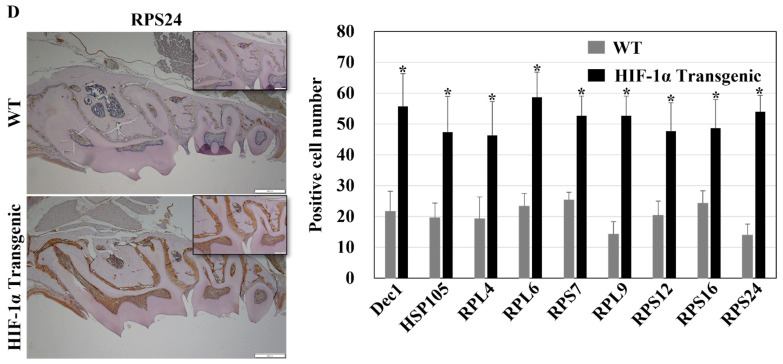
HIF-1α facilitates Dec1 expression and ribosomal protein regulation in mouse tissues. (**A**–**D**) Immunohistochemical staining showing the expression levels of Dec1, HSP105, and ribosomal proteins in the PDL of WT and HIF-1α transgenic mice. Scale bars = 500 μm. All results are representative of at least three independent experiments. The data shown represent means ± SD; * *p* < 0.05.

The distribution and localization of Dec1, HSP105, and ribosomal proteins were analyzed in HIF-1α transgenic mice. Histological analysis showed a similar morphology in WT mice and in HIF-1α transgenic mice (Figure 5A). The expression of Dec1, HSP105, RPL4, RPL6, RPS7, RPL9, RPS12, RPS16, and RPS24 was higher in PDL fibroblasts of HIF-1α transgenic mice compared to WT mice (Figure 5A–D).

### 2.6. OTM Elicits High Dec1 Expression in PDL Fibroblasts of WT Mice

**Figure 6:**
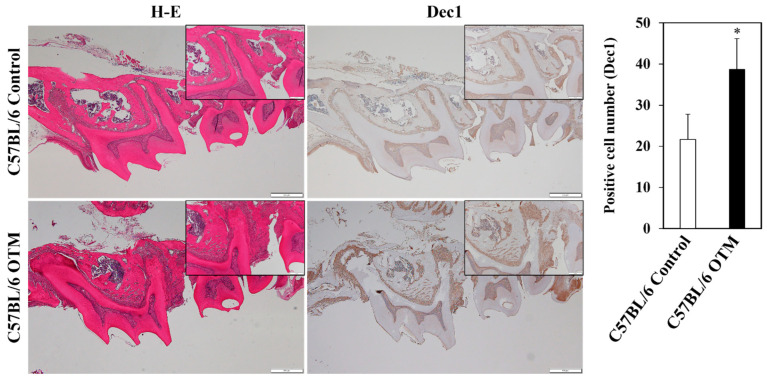
Dec1 is induced in the OTM of WT mouse tissues. Immunohistochemical staining revealed a higher expression of Dec1 in WT OTM mice compared to the control. Scale bars = 500 μm. All results are representative of at least three independent experiments. The data shown represent means ± SD; * *p* < 0.05.

To analyze the involvement of Dec1 in OTM, an experimental model of OTM in WT mice was employed. Representative H-E-stained images showed changes in the distance between the first and second molars, thus displaying superior tooth movement in the OTM model. Dec1 expression was markedly increased in the PDL fibroblasts of WT OTM mice compared to the control (Figure 6).

### 2.7. A Deficiency of Dec1 Attenuates OTM in Mice

**Figure 7:**
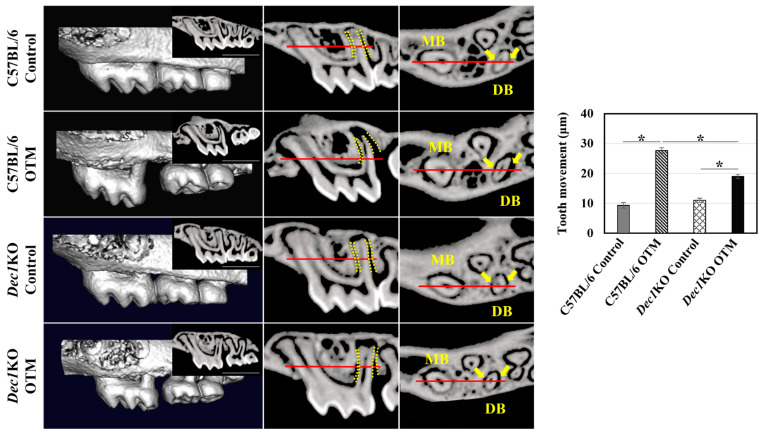
OTM and PDL width measurements. 3D images showing that the distance between the first and second molars in the WT OTM mice is much wider than in the *Dec1*KO OTM mice. OTM was quantified by measuring the minimum distance between the maxillary first and second molar crowns. Scale bars = 1 mm. The average PDL width was quantified by measuring the mean PDL space on the distal aspect of the distobuccal root in sagittal micro-CT images. The mid-root PDL width was measured in the horizontal micro-CT images. MB, mesiobuccal root; DB: distobuccal root. The data shown represent the means ± SD of at least three independent experiments; * *p* < 0.05.

To further confirm the involvement of Dec1 in tooth movement, *Dec1*KO mice were generated, followed by analysis using the experimental OTM mouse model. µCT analysis revealed that the tooth movement between the maxillary first and second molars was considerably lower in *Dec1*KO mice compared to WT mice (Figure 7).

### 2.8. Reduced Expression of Ribosomal Proteins in PDL Fibroblasts of Dec1KO OTM Mice

**Figure 8:**
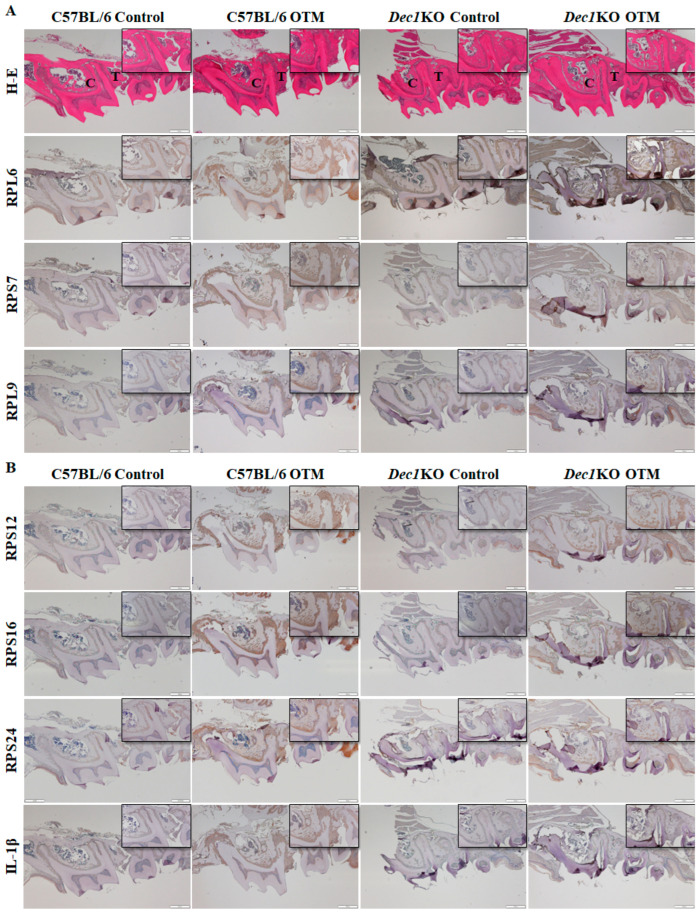

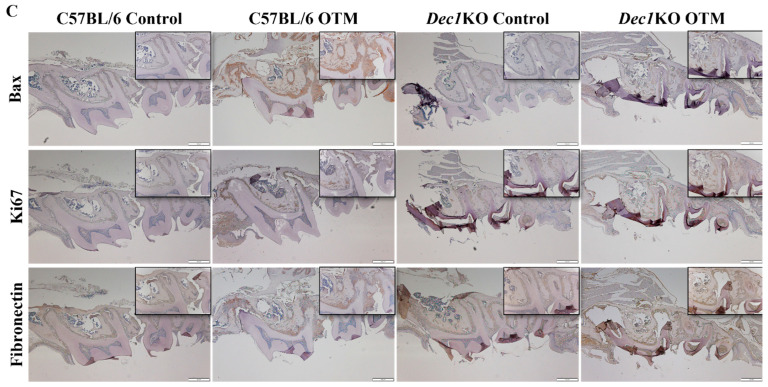
Dec1 deficiency attenuates tooth movement, ribosomal protein regulation, and inflammation in the OTM mouse model. (**A**) H-E staining of WT OTM mice shows a wider distance between the first and second maxillary molars compared to *Dec1*KO mice during orthodontic treatment. (**A**,**B**) Expression levels of ribosomal proteins and IL-1β were decreased in *Dec1*KO OTM mice compared with WT OTM mice. Scale bars  =  500 μm. All results are representative of at least three independent experiments. (**C**) Bax expression was decreased in *Dec1*KO OTM mice compared with WT OTM mice. Ki67 and fibronectin expression were increased in *Dec1*KO OTM mice. Scale bars  =  500 μm. All results are representative of at least three independent experiments. C, compression. T, tension.

**Figure 9:**
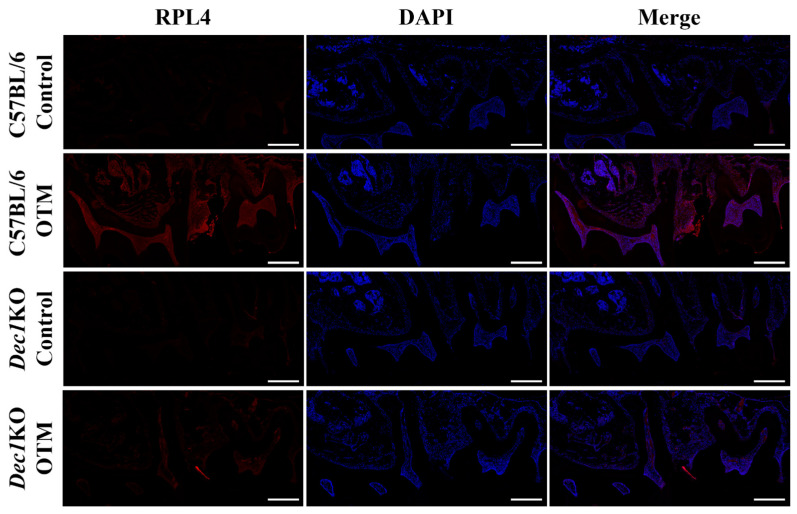
A deficiency of Dec1 reduces the expression of RPL4 in the OTM mouse model. Immunofluorescence analysis shows that the expression level of RPL4 decreased in *Dec1*KO OTM mice compared with WT OTM mice. Scale bars  =  100 μm. All results are representative of at least three independent experiments.

Immunohistochemical staining was used to determine the expression patterns of ribosomal proteins in the PDL fibroblasts. Interestingly, the degree of staining of RPL6, RPS7, RPL9, RPS12, RPS16, and RPS24 was markedly increased in WT OTM mice compared to *Dec1*KO OTM mice (Figure 8A,B). Dec1-deficient mice showed decreased expression of IL-1β in the OTM model (Figure 8B). Bax protein expression was decreased in *Dec1*KO OTM mice compared with WT OTM mice. Ki67 and fibronectin expression were increased in *Dec1*KO OTM mice (Figure 8C). Immunofluorescence staining (Figure 9) revealed that WT OTM mice had increased expression of RPL4 in PDL fibroblasts compared to *Dec1*KO OTM mice.

### 2.9. Dec1 Is Required for Inflammatory and Heat Shock Responses during OTM

**Figure 10:**
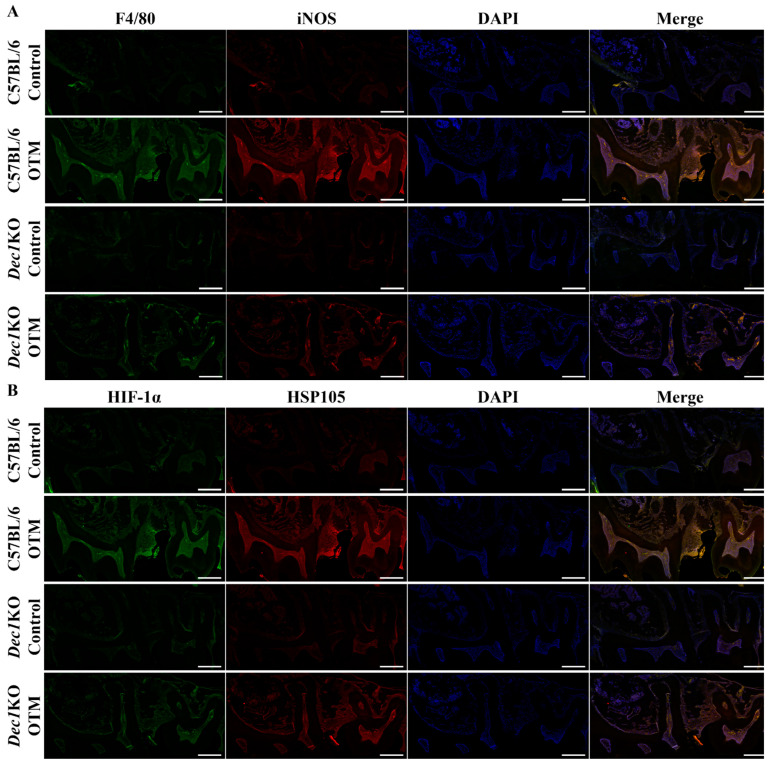
Effect of Dec1 on F4/80, iNOS, HIF-1α and HSP105 in the OTM mouse model. (**A**) F4/80 and iNOS were reduced in the PDL fibroblasts of *Dec1*KO OTM mice compared with WT OTM mice. (**B**) Deficiency of Dec1 decreased the expression of HIF-1α and HSP105 in PDL fibroblasts of the OTM mouse model. All specimens were incubated with Zenon labeling complex and mounted with ProLong™ Gold Antifade Mount with DAPI. Scale bars = 100 μm. All results are representative of at least three independent experiments.

Next, the role of Dec1 in inflammation and the regulation of HSPs was determined in OTM mice. Double-immunofluorescence staining showed that the expression of F4/80 and iNOS were both induced in PDL fibroblasts in WT OTM mice compared to *Dec1*KO mice, thus validating the importance of Dec1 in inflammatory responses during OTM (Figure 10A). HIF-1α and HSP105 were also highly expressed in WT OTM mice, but were decreased in *Dec1*KO mice (Figure 10B).

### 2.10. Compression Force Induces Hypoxia-Responsive Genes, Heat Shock Proteins, Ribosomal Proteins, and Inflammatry Molecules in hPDL Fibroblasts

**Figure 11:**
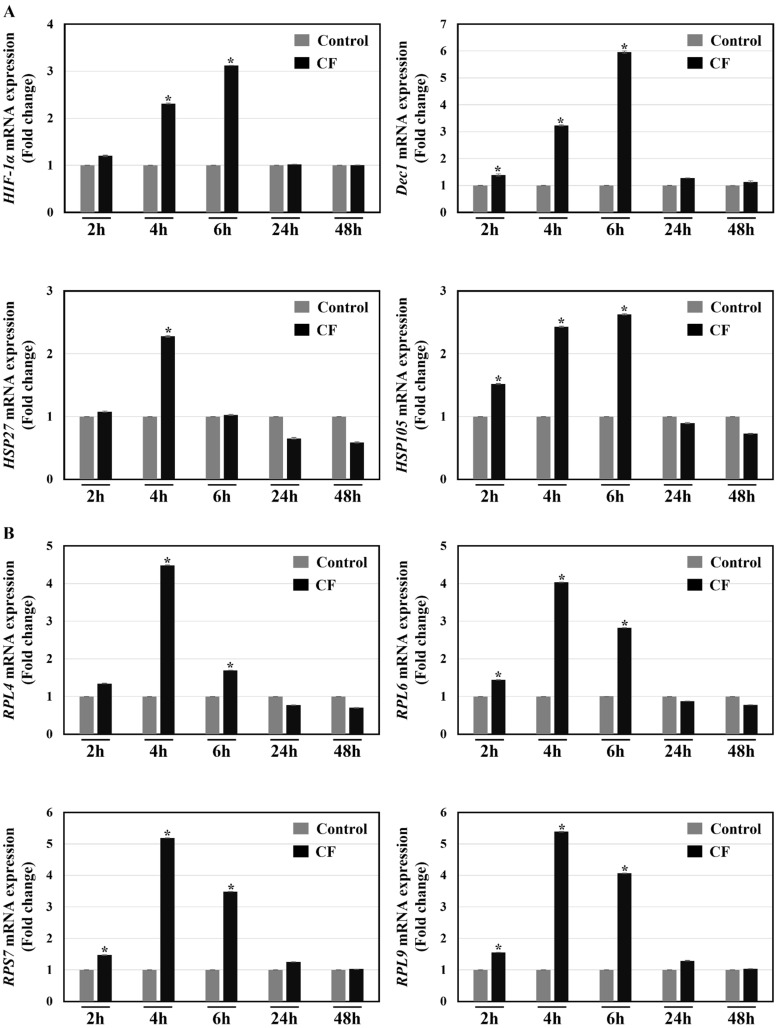

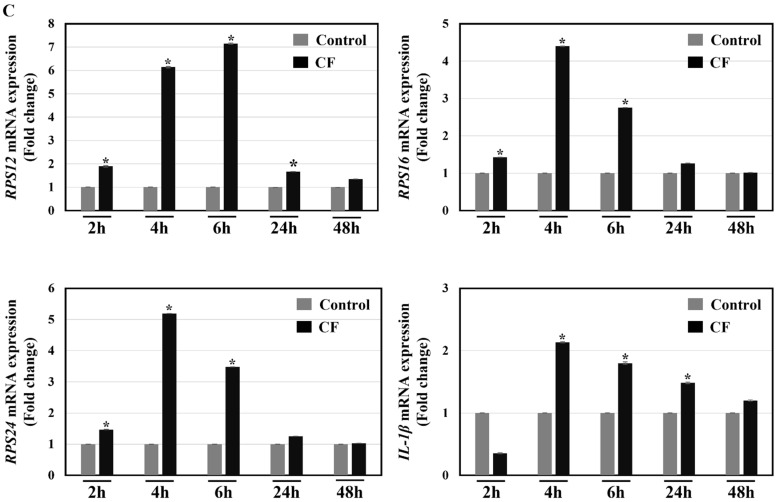
Regulation of hypoxia-responsive genes, heat shock proteins, and ribosomal proteins in hPDL fibroblasts under compression force (CF). (**A**) Quantitative RT-PCR analysis reveals that the expression levels of HIF-1α, Dec1, HSP27, and HSP105 are induced at 4 and 6 h in the CF group. (**B**) CF treatment results in the induced expression of RPL4, RPL6, RPS7, and RPL9 in hPDL fibroblasts. (**C**) The expression levels of RPS12, RPS16, RPS24, and IL-1β were increased at 4 and 6 h in the CF group compared to the control group. The data shown represent means ± SD; * *p* < 0.05. All results are representative of experiments done in triplicate. CF, compression force.

To characterize the regulation of heat shock responses and the expression of ribosomal proteins under a compression force environment, human PDL fibroblasts were subjected to a 2.0 g/cm^2^ compression force for different times. qRT-PCR analyses showed that most genes exhibited significant increases at 2, 4, and 6 h of treatment (Figure 11A–C). Treatment with the compression force for 24 and 48 h did not show significant changes compared to the control (Figure 11A–C). The protein expression level of RPS12 was significantly decreased in compression force-treated hPDL fibroblasts at 24 and 48 h (Supplementary Figure S1). The RPS16 protein showed a significant increase at 24 h after compression force treatment (Supplementary Figure S1). The expression of RPS24 protein was significantly downregulated at 48 h, while Bax showed the opposite in compression force-treated hPDL fibroblasts at 48 h (Supplementary Figure S1). The Dec1 protein expression level was significantly upregulated at 24 h and decreased thereafter (Supplementary Figure S1). No significant changes were observed in the expression of other ribosomal proteins and HIF-1α protein (Supplementary Figure S1).

### 2.11. Silencing of Dec1 Abrogates the Expression of Ribosomal Proteins, Apoptosis, Migration, and Inflammation in PDL Fibroblasts under a Hypoxia Environment

**Figure 12:**
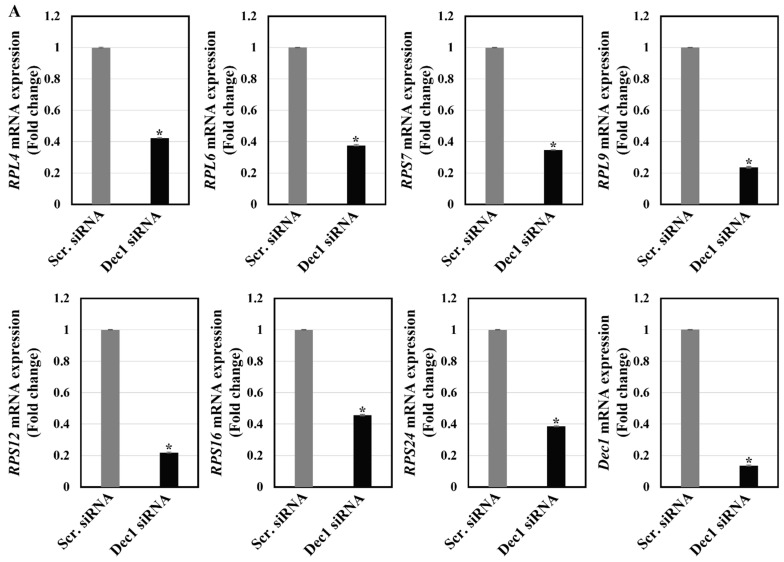

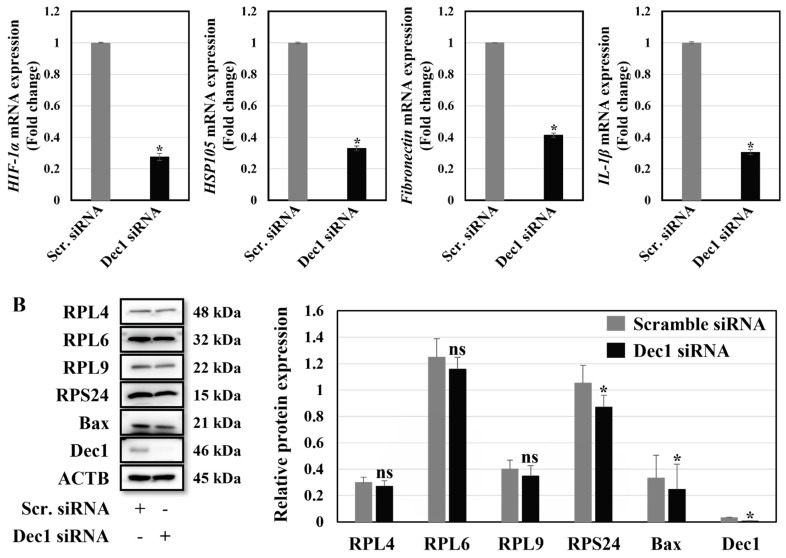
Dec1 knockdown inhibits the expression of ribosomal proteins in hPDL fibroblasts in a hypoxia environment. (**A**) Quantitative RT-PCR analysis reveals that the expression levels of ribosomal proteins, HIF-1α, HSP105, fibronectin and IL-1β are reduced in Dec1 siRNA transfected hPDL fibroblasts. The data shown represent means ± SD; * *p* < 0.05. All results are representative of experiments done in triplicate. (**B**) Western blot analyses reveal the suppressed expression of RPL4, RPL6, RPL9, RPS24, and Bax in the Dec1 siRNA transfected hPDL fibroblasts. The data shown represent the means ± SD of experiments carried out in triplicate; * *p* < 0.05. ns, not significant.

To corroborate the role of Dec1 in the hypoxic regulation of ribosomal proteins, apoptosis, migration, and inflammation, PDL fibroblasts were transfected with a scrambled siRNA or a Dec1 siRNA. qRT-PCR analysis demonstrated a significant inhibition of all ribosomal proteins, HIF-1α, HSP105, fibronectin and IL-1β, in the Dec1 knockdown group (Figure 12A). Western blot analysis revealed a significant inhibition of RPS24, and Bax expression in Dec1 siRNA transfected PDL fibroblasts (Figure 12B).

### 2.12. The Wound Healing Process Is Accelerated by a Dec1 Deficiency

**Figure 13:**
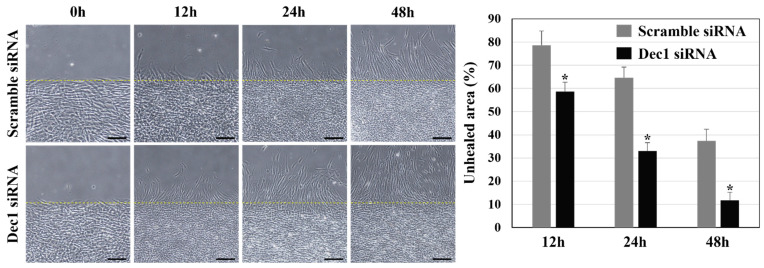
*Dec1* deficiency promotes the migration of hPDL fibroblasts under hypoxic conditions. The cell migration ability of hPDL fibroblasts is induced by Dec1 knockdown at all time points, compared with the control group. Scale bars = 20 µm. The data shown represent the means ± SD of experiments carried out in triplicate; * *p* < 0.05.

To further verify the effects of Dec1 on the wound healing process, the cell motility of hPDLFs was evaluated in wound healing assays after transfection of hPDL fibroblasts with Dec1 siRNA under hypoxic conditions. As shown in Figure 13, compared with the control group, cell migration ability was significantly increased in hPDL fibroblasts at all time points after Dec1 knockdown in the experimental group.

## 3. Discussion

Increased knowledge about the cellular regulatory mechanisms of PDL fibroblasts responding to mechanical forces is required to develop effective therapies in orthodontics. Determining gene expression profiles during orthodontic force application and the differential gene expression patterns that result will contribute to understanding the main drivers for refining orthodontic treatments and improving their efficiency in OTM.

Analysis of large data sets is a daunting task. Bioinformatics resources are intended to identify the biological potential of DEGs [31]. The limited number of complete gene expression profiles and the large differences in the methodologies used make it difficult to interpret the results. Considering the hypoxia-responsive tissue profile, the analysis was extended to the hypoxic regulation of PDL fibroblasts. In this study, all genes that were markedly related to hypoxia were selected by WGCNA analysis of fibroblasts based upon two co-expression modules. Gene ontology enrichment was applied to determine the core biological processes that are connected to the DEGs. KEGG pathway enrichment analyses were also carried out (Supplementary Table S2), and ribosomes were identified as the most enriched biological process.

Ribosomal biogenesis is an integrated system that involves ribosomal protein production, and this system is intricately connected to cell growth and proliferation [32]. Ribosomal proteins preserve a balance between protein synthesis and other biological functions, and they are engaged in distinct cellular physiological processes. RPL4 interacts with c-Myb to regulate c-myc expression [33]. RPL6 elevates cyclin E expression to induce cell proliferation in gastric cancer [22]. Recent research has demonstrated that a disturbance in ribosome biogenesis results in ribosomal stress [26]. In terms of ribosomal stress, RPS7 is involved in p53-dependent apoptosis and cell cycle arrest [34]. RPL9 functions as an antiapoptotic gene that leads to enhanced stress-mediated survival [35]. RPS12 initiates the regulation of translation in hypoxia [36]. RPS16 is also concerned with apoptosis and erythrocyte differentiation under hypoxic conditions [37]. The alternative splicing of RPS24 depends on hypoxia [38]. In this study, the rRNA transcription of processing was not changed in the control and experimental groups (data not shown); hence, the importance of extra ribosomal factors is a prerequisite. Our findings support the concepts of those published reports, which in turn assists in understanding the integral functions of ribosomal proteins regarding cellular stresses.

HSPs are highly conserved proteins that are expressed in response to stress conditions such as heat shock and hypoxia [39]. HIF-1α activates HSP27 in hypoxic ischemic insults [40]. Muraoka et al. demonstrated HSP27 expression and phosphorylation in the PDL fibroblasts of mice after orthodontic force application [18]. HSP27 overexpression also enhances collagen synthesis [41]. HSP105 regulates the accumulation and transcriptional activation of HIF-1α [42]. Our study reveals the enhanced expression of HSPs in the experimental group compared to the control group at day 14, which is attributed to the maintained expression of ribosomal proteins. The reduction in mRNA levels may be regarded as alleviating HSP expression when a tissue attains a homeostatic level. Our study implies that HSPs have been strongly associated with maintaining the homeostasis of PDL fibroblasts. To this end, the current findings expand our understanding of periodontal remodeling and validate the special emphasis aimed at the cytoprotective activities of HSPs, indicating that they are principal modulators of PDL fibroblasts in a hypoxic environment during OTM.

The transcription factor HIF-1α represents the master regulator of cellular adaptation to hypoxia and contributes to the various pathophysiological implications of hypoxia [43]. HIF-1α is the first host response in the bone remodeling process and in OTM [44]. The levels of Dec1 are considerably increased in cells cultured in a hypoxic environment. The functional hypoxia-responsive element in Dec1 binds to HIF-1α, providing a biological connection to the transcriptional regulation of Dec1 under hypoxia [13]. Our group previously demonstrated the intervention of PI-3K signaling in Akt phosphorylation and Dec1 overexpression [45]. Dec1 restrains cyclin D1 gene transcription [46] and the deletion of Dec1 prevents cardiac dysfunction against fibrosis, inflammation, and myocardial stromal cell apoptosis [47]. In this study, the loss of Dec1 facilitated the proliferation and migration and inhibited inflammation and apoptosis of PDL fibroblasts under a hypoxic environment. The results from our experiments showed that the mechanotransduction and hypoxic environment of PDL fibroblasts play a leading role in the intervention of OTM. Orthodontic loading drives local hypoxia of the PDL, originating as an inflammatory cascade [8]. To this end, our data indicate that the ablation of Dec1 may be a useful approach to suppress the excessive expression of IL-1β, F4/80 and iNOS. Our results indicate that hypoxia induced in periodontal tissue is among the factors involved in the similar expression paradigms of Dec1 and ribosomal proteins during OTM. Our results demonstrate that an orthodontic force provokes hypoxic and inflammatory responses in vivo and in vitro, generating ribosomal protein dysregulation. The present results offer a conceptual basis for fostering periodontal tissue homeostasis during OTM. Nevertheless, the comprehensive functional analysis of the Dec1 regulation of ribosomal proteins should assume the involvement of specific intricate signaling pathways.

The results of this study demonstrate that Dec1 is instrumental in the genetic control of OTM. The functional involvement of Dec1 in accelerating tooth movement was moderately assigned to enhanced ribosomal protein expression in PDL fibroblasts, illustrating that the intercellular crosstalk endured between Dec1 and ribosomal proteins. Interestingly, the knockdown of Dec1 in fibroblasts or Dec1 inhibition significantly reduced ribosomal protein expression in this study, which implies that inhibiting Dec1 could indeed block ribosomal protein expression or might hinder OTM by decreasing ribosomal protein expression. These results reveal for the first time that Dec1 is involved in responding to mechanical stress and integrated downstream ribosomal protein expression during OTM. Thus, our study delivers fresh perspectives on the role of Dec1 in regulating the expression of ribosomal proteins during OTM.

OTM transpires by virtue of various mechanisms involving mechanotransduction [4], local hypoxia [44] and sterile inflammation [8]. This basic scientific research approach could help decipher the fundamentals of orthodontic movements, such as using clear aligners as an aesthetic option for orthodontic treatment. A recent study demonstrated that prototype orthodontic models are precise and satisfactory as a diagnostic approach for the indirect fabrication of clear aligners [48]. Thus, there is an urgent need to characterize the ribosomal stress induced at different intensities of programmed movement for each type of aligner, since clear aligners are unable to evade external root resorption [49]. Furthermore, low-level laser therapy (LLLT) triggers OTMs, advancing alveolar bone turnover, and LLLT makes treatments with invisible removal aligners more comfortable [50]. The authors surmise that it would be of clinical interest to investigate OTMs with the use of bio-stimulation systems.

Fibroblasts or immune cells of the PDL are vital regulators in OTM that govern the range of bone remodeling by generating proteins and inflammatory cytokines that influence osteoclastogenesis, consequently regulating OTM [51]. Our study is the first to demonstrate a connection between orthodontic force, Dec1, and ribosomal proteins in periodontal fibroblasts. Our in vitro and in vivo studies show that Dec1 and ribosomal proteins are constantly influenced by mechanical loading. The result that Dec1 and ribosomal proteins appear to have core functions in the continued and balanced alteration of mechanically stressed cells and in resistance against mechanical overload delivers a unique perception into the mechanotransduction avenues of PDL fibroblasts. In this study, the poor correlation between the level of mRNA and the level of protein may be due to the efficiency of protein translation affected by microenvironmental stimuli. Our findings demonstrate the significance of Dec1 and ribosomal proteins in PDL fibroblasts and demonstrate their influence on mechanical stress defense. These findings build a foundation for a fundamental understanding of the intricate regulatory events involved in physiopathological forces applied to the PDL. Such features, in conjunction with future research into the link between Dec1 and ribosomal proteins in the regulation of mechanical force, will assist in understanding the biological processes caused by OTM.

Animal models are employed to corroborate in vitro results and to ascertain the latent beneficial effects of Dec1 and ribosomal proteins. Male mice were used in this study to eliminate any potential effects of estrogen. The authors confirmed the idea of a *Dec1*KO mouse model to explore the possible cytoprotective effects of mechanical stress in PDL fibroblasts. These results reveal for the first time that experimental tooth movement is delayed in *Dec1*KO mice. Micro-CT analysis indicated that WT mice show the highest movement of teeth compared to *Dec1*KO mice during orthodontic treatment, providing evidence that Dec1 might alleviate tooth movement. One explanation for this is that *Dec1*KO mice are less sensitive to mechanical strains, as suggested in our previous report on periodontal inflammation [52]. A deficiency of Dec1 might thus be a feasible therapeutic method for diminishing orthodontic treatment-related periodontal risk in the future, which must be addressed by more comprehensive analysis. To best elucidate the function of Dec1 in tooth movement, local or systematic application of Dec1 proteins or Dec1 monoclonal antibodies during OTM must be carried out in the future to examine whether Dec1 regulates bone remodeling during tooth movement.

## 4. Materials and Methods

### 4.1. Download and Processing of Single-Cell Sequencing Data

The GEO database was employed to obtain the single-cell sequencing data set GSE164157 of human teeth. Data quality control was then executed for a total of 5 samples comprising the data set. The following criteria were adopted in this analysis: cells with 10% or less expression of mitochondrial genes, cells with more than 200 genes expressed in at least three types of cells, and 200 to 7000 range of gene expression levels in at least three types of cells. Three thousand highly variable genes were selected. SCT correction [53] was applied to integrate the data. The “dims” parameter was set at 20, the data dimension was reduced using the tSNE method [54], and the clustering of cells was carried out at a resolution of 1.0 using the “KNN” method [55]. Thereafter, cells were characterized by several cell surface-specific markers.

### 4.2. Single Sample Gene Set Enrichment Analysis (ssGSEA)

The GSEA website (https://www.gsea-msigdb.org/gsea/index.jsp, accessed on 24 May 2022) was used to download the hypoxia gene set. ssGSEA analysis was performed to obtain scores linked to hypoxia in fibroblasts.

### 4.3. Weighted Co-Expression Network Analysis (WGCNA)

WGCNA was carried out to discover the gene modules corresponding with hypoxia in fibroblasts, and genes associated with hypoxia were identified.

### 4.4. Animals

Dec1-knockout (*Dec1*KO) and HIF-1α transgenic mice were used, as described in our previous studies [52,56]. In brief, the Ingenious Targeting Laboratory, Inc. (Stony Brook, NY, USA) generated *Dec1*KO mice, setting out a Neo cassette that substituted the full coding sequences in exons 1–5 of Dec1. The chimeras were backcrossed for 3 generations with C57BL/6J mice. Male *Dec1*KO mice (*n* = 6), HIF-1α transgenic mice (*n* = 6), and wild-type (WT) littermates (*n* = 6) each for KO and transgenic mice were housed under specific pathogen-free conditions. The mice were sacrificed by anesthesia, followed by cervical dislocation. All animal experiments were approved by the Animal Care and Use Committee of Hiroshima University (A20-116) and the Nihon University School of Dentistry at Matsudo (AP20MAS016-1, AP21MAS011-1).

### 4.5. OTM Model

Orthodontic force was applied as follows: briefly, a nickel-titanium coiled spring (wire size, 0.2 mm; diameter and length, 1 mm; Tomy International, Inc., Tokyo, Japan) was placed between the incisor and the left maxillary first molar of each mouse, thus providing a force of 30–35 g [29] in WT and in *Dec1*KO mice, and a flowable restorative resin (3M ESPE, Minneapolis, MN, USA) was applied to bond the spring. The mice had ad libitum access to soft food and water and were sacrificed on day 14 post-induction.

### 4.6. Micro-Computed Tomography (Micro-CT)

Micro-CT (R_mCT2, Rigaku Corp., Tokyo, Japan) was employed to capture three-dimensional (3D) images of the upper jaws of the mice under the following exposure conditions: tube voltage, 90 kV; tube current, 200 μA; voxel size, 20 × 20 × 20 μm.

### 4.7. Histological Analysis, Immunohistochemistry, and Immunofluorescence

The jaw tissues of the mice were fixed in 4% paraformaldehyde at 4 °C for 48 h, decalcified in 10% EDTA-2Na pH 7.0 (Muto Pure Chemical Co., Ltd., Tokyo, Japan) for 5 days, and then embedded in paraffin. Jaw tissue sections were stained with hematoxylin-eosin (H-E) (Muto, Tokyo, Japan) for histopathological observations with a light microscope. Four μm tissue sections of WT, HIF-1α transgenic and *Dec1*KO mice were incubated in 10x citrate buffer, pH 6.0 (Abcam, Tokyo, Japan), at 95 °C for 10 min. The sections were then immersed in peroxidase blocking solution (DAKO, Carpinteria, CA, USA) for 10 min, followed by a 1 h incubation in protein block solution (DAKO, Carpinteria, CA, USA). The sections were then incubated overnight with primary antibodies from Abcam (Cambridge, UK) [(RPL4, ab174269, 1:100), (RPL9, ab182556, 1:100), (RPS12, ab167428, 1:100), (RPS16, ab177951, 1:100), (RPS24, ab196652, 1:50), (HSP105, ab109624, 1:100), (IL-1β, ab9722, 1:100), (Bax, ab32503, 1:100), (Ki67, ab16667, 1:100), (Fibronectin, ab45688, 1:200)], from Thermo Fisher Scientific (Waltham, MA, USA) [(RPL6, PA5-30217, 1:100), (RPS7, PA5-110326, 1:150)], and from Novus Biologicals (Centennial, CO, USA) [(Dec1, NB100-1800, 1:100)], followed by incubation with appropriate secondary antibodies (MAX-PO, Nichirei Biosciences Inc., Tokyo, Japan) at room temperature for 30 min. For immunohistochemistry, diaminobenzidine (DAB) solution (DAKO, Carpinteria, CA, USA) was employed to develop color, and hematoxylin counterstaining was performed. An optical microscope (DP74; Olympus, Tokyo, Japan) was used to obtain the images. The stained cells were photographed, and four randomly selected microscopic fields per specimen were quantified using a x40 objective lens (0.2 mm^2^). The ratio of positive stained area to total area was calculated. For immunofluorescence, all specimens were incubated with antibodies from Abcam (Cambridge, UK) [(F4/80, ab111101, 1 μg), (iNOS, ab178945, 1 μg), (HIF-1α, ab2185, 1 μg)] and Zenon labeling complex and mounted with ProLong™ Gold Antifade Mount with DAPI (Thermo Fisher Scientific, Waltham, MA, USA). For imaging of the entire sagittal section of upper jaw (three molar teeth: M1, M2, and M3), the sections were imaged as a Z-stack under an all-in-one fluorescence microscope (BZ-X800, KEYENCE, Osaka, Japan) equipped with an advanced observation module (BZH4XD, KEYENCE, Osaka, Japan) and a Plan Apochromat 20x objective (NA0.75, BZ-PA20, KEYENCE, Osaka, Japan) was used to capture tiled images, and the image stitching function in the BZ-X Analyzer software (BZ-H4A, KEYENCE, Osaka, Japan) was used for image stitching.

### 4.8. Cell Culture

Human periodontal ligament (hPDL) fibroblasts were purchased (Cat# CC-7049, Lot# 0000571477, Lonza, Tokyo, Japan). SingleQuots supplements (CC-4181, insulin, hFGF-beta, GA-1000, and FBS) were added to the culture medium. Cells at 80–85% confluence were subcultured, and cells at passages 5–6 were employed for subsequent analyses. Pre-incubation of cells in culture medium containing 1% FBS was performed for 16 h. A slide glass was then placed over the cells in 6-well plates and were subsequently exposed to 2.0 g/cm^2^ of compression force for 2, 4, 6, 24, and 48 h and then subjected to quantitative Real-Time PCR (qRT-PCR) analysis. Western blot analysis was carried out using the 24 and 48 h samples (Supplementary Figure S1).

### 4.9. siRNA Transfection

About 1.2 × 10^5^ hPDL fibroblasts were cultured in 6-well plates in antibiotic-free medium for 16 h. RNAiMAX (Thermo Fisher Scientific, Waltham, MA, USA) was used to transfect cells with a negative control siRNA or a Dec1 siRNA for 48 h. Cells were then placed in a sealed airtight box containing an AnaeroPack (Mitsubishi Gas Chemical Co., Inc., Tokyo, Japan) to maintain a hypoxic environment (<1%) during transfection. Cell lysates were then collected and used for qRT-PCR and Western blot analyses.

### 4.10. RNA Extraction and qRT-PCR

A miRNeasy Mini Kit (Qiagen KK, Tokyo, Japan) was used to extract total RNA from hPDL fibroblasts. A 1 μg aliquot of each RNA was synthesized and transcribed to cDNA using a SuperScript VILO cDNA Synthesis Kit (Thermo Fisher Scientific, Waltham, MA, USA). TaqMan probes (all from Thermo Fisher Scientific, Waltham, MA, USA) for RPL4 (Hs03044646_g1), RPL6 (Hs03044365_g1), RPS7 (Hs00962846_gH), RPL9 (Hs01552541_g1), RPS12 (Hs00831630_g1), RPS16 (Hs01598518_gH), RPS24 (Hs03006009_g1), Dec1 (Hs00186419_m1), HIF-1α (Hs00153153_m1), HSP27 (Hs00356629_m1), HSP105 (Hs00971475_m1), IL-1β (Hs00174097_m1), Fibronectin (Hs00277509_m1), and ACTB (Hs99999903_m1) were used for qRT-PCR. The QuantStudio 7 Flex Real Time PCR System (Thermo Fisher Scientific, Waltham, MA, USA) was used for qRT-PCR and ExpressionSuite v1.3 Software was employed to quantify relative gene expression.

### 4.11. Western Blot Analysis

RIPA lysis buffer (Santa Cruz Biotechnology, Santa Cruz, CA, USA) and a BCA Protein Assay Kit (Pierce Biotechnology, Rockford, IL, USA) were used to lyse cells and to measure protein concentrations, respectively. Twenty μg protein per lane was separated by electrophoresis followed by blotting to PVDF membranes. The membranes were incubated overnight with primary antibodies: from Abcam (Cambridge, UK) [(RPL4, ab174269, 1:1000), (RPL9, ab182556, 1:2000), (RPS12, ab167428, 1:1000), (RPS16, ab177951, 1:1000), (RPS24, ab196052, 1:1000), (HSP105, ab109624, 1:1000), (Bax ab32503, 1:1000), (HIF-1α, ab2185, 1:500)], from Thermo Fisher Scientific (Waltham, MA, USA) [(RPL6, PA5-30217, 1:1000), (RPS7, PA5-110326, 1:1000)], from Novus Biologicals (Centennial, CO, USA) [(Dec1, NB100-1800, 1:1000)] and from Cell Signaling Technology (Danvers, MA USA) [β-actin, 4970, 1:1000), (GAPDH, 2118, 1:1000) followed by incubation with horseradish peroxidase-conjugated anti-rabbit IgG (1:2000; Cell Signaling Technology, Danvers, MA, USA) at room temperature for 1 h. An ECL Plus Western Blotting Detection System (GE Healthcare, Tokyo, Japan) was used to visualize the immunoreactive bands, and images were captured using the Luminescent ImageQuant LAS 4000 Mini (GE Healthcare, Tokyo, Japan). Densitometric analyses were performed with Image J software.

### 4.12. Wound Healing Assay

hPDL fibroblasts were harvested after transfection with a scrambled siRNA or a Dec1 siRNA under hypoxic conditions from monolayer cultures at 80% confluence by brief trypsinization and were seeded in Ibidi wound healing dishes (Ibidi, Bavaria, Germany) where they were grown to confluence in serum-free medium for 24 h. The insert walls of the Ibidi wound healing dishes were removed after 24 h, and the migration of hPDLFs into scraped areas was observed and photographed at 12 h, 24 h, and 48 h. An inverted light microscope with a grid reticle in the microscope ocular was used to measure the closure of the gap, which was measured at six marked sites. The percentage of the starting distance at the wound edges was set to determine the unhealed areas. All results are representative of experiments conducted in triplicate. Data from these independent experiments are reported as means ± standard deviation.

### 4.13. Statistical Analysis

The Statistical Package for the Social Sciences (SPSS) 19.0 software was used to perform statistical analyses. An independent two-tailed Student’s *t*-test or analysis of variance (ANOVA) was used to assess the quantitative variables. *p* values < 0.05 are statistically significant.

## 5. Conclusions

In conclusion, there is a consensus from all our experiments that mechanical force applied to PDL cells leads to a modified release of HSPs and inflammatory molecules, which then results in an altered initiation of the hypoxic response, as demonstrated by the induced expression of HIF-1α and Dec1, which then increases ribosomal protein expression.

## Data Availability

The datasets used and analyzed in this study are available from the corresponding author (bhawal.ujjal.kumar@nihonu.ac.jp) on reasonable request.

## Supplementary Materials

The following supporting information can be downloaded at: https://www.mdpi.com/article/10.3390/ijms24010618/s1.
