# Supplementary material for: Ribosomal Stress Couples with the Hypoxia Response in Dec1-Dependent Orthodontic Tooth Movement
Source: Int J Mol Sci. 2022 Dec 29;24(1):618. doi: 10.3390/ijms24010618 (PMC9820322; doi:10.3390/ijms24010618)
Supplement: Supplementary file 1 [file ijms-24-00618-s001.zip › Supplementary Figure S1.pdf]

Supplementary Results

Ribosomal protein expression was examined in compression force (CF) treated hPDL fibroblasts for 24 h and 48 h. The protein expression level of RPS12 was significantly decreased in CF treated hPDL fibroblasts at 24 h and 48 h. RPS16 protein showed a significant increase at 24 h after CF treatment. The expression of RPS24 protein was significantly downregulated at 48 h while Bax showed the opposite in CF treated hPDL fibroblasts at 48 h. Dec1 protein expression level was significantly upregulated at 24 h and decreased thereafter. There were no significant changes observed in other ribosomal protein and HIF-1α expression.

Supplementary Figure Legend

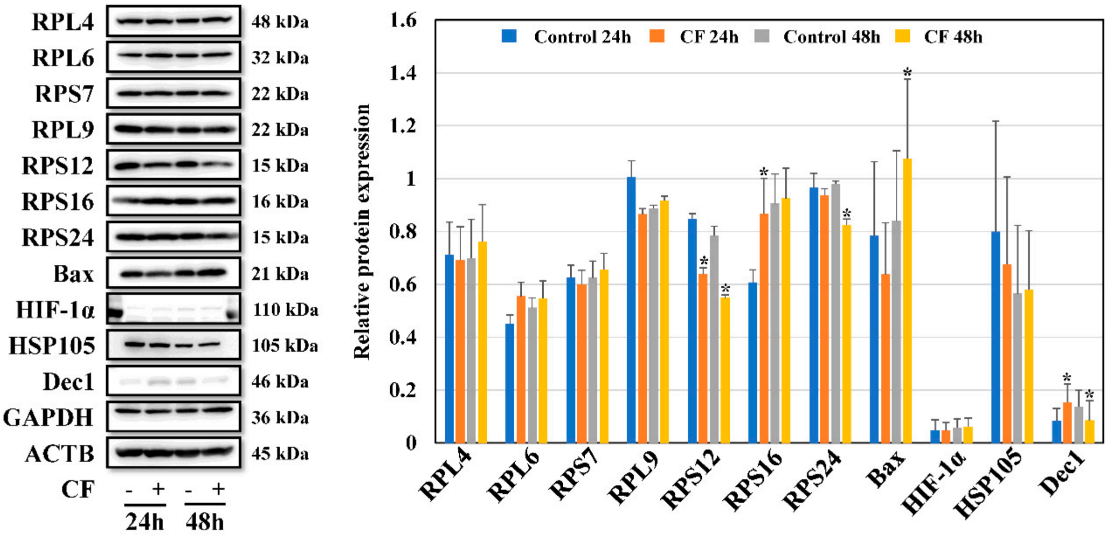

Supplementary Figure S1. Western blot analysis presented changes in ribosomal protein expression at 24 h and 48 h after CF treatment in hPDL fibroblasts compared to the controls. All results are representative of experiments done in triplicates. CF, compression force. * $p < 0.05$.
