# Supplementary material for: Ribosomal Stress Couples with the Hypoxia Response in Dec1-Dependent Orthodontic Tooth Movement
Source: Int J Mol Sci. 2022 Dec 29;24(1):618. doi: 10.3390/ijms24010618 (PMC9820322; doi:10.3390/ijms24010618)
Supplement: Supplementary file 1 [file ijms-24-00618-s001.zip › Supplementary Table S1.pdf]

Supplementary Table S1: Surface marker genes of different cell types

cell_type	cell_marker		
MSCs	FRZB	1	mesenchymal stem cells
MSCs	NOTCH3		
MSCs	MYH11		
MSCs	THY1		
Fibroblasts	MDK	4	
Fibroblasts	COL1A1		
ECs	EDN1		endothelial cells
ECs	CLDN5	6	
ECs	CXCLI2		
ECs	ACKR1	3	
ECs	CD234	7	
ECs	INSR		
ECs	RGCC		
ScCs	SOX10	10	Schwann cells
ScCs	GFRA3		
ScCs	NGF		
ScCs	NGFR		
Immune Cells	PTPRC	5	
Immune Cells	CD3E	9	
Immune Cells	CD3D		
Immune Cells	MZB1	8	
Immune Cells	CSF1R		
Epithelial	KRT14		
Epithelial	ODAM	2	
Epithelial	WNT10A	0	
Epithelial	IL1A		
Epithelial	IL1B		
Erythrocytes	HBB		
