# Supplementary material for: Ribosomal Stress Couples with the Hypoxia Response in Dec1-Dependent Orthodontic Tooth Movement
Source: Int J Mol Sci. 2022 Dec 29;24(1):618. doi: 10.3390/ijms24010618 (PMC9820322; doi:10.3390/ijms24010618)
Supplement: Supplementary file 1 [file ijms-24-00618-s001.zip › Supplementary Table S2.pdf]

Supplementary Table S2: Signaling pathways and molecules obtained from bioinformatics analysis

Signaling pathways	Molecules
**TNF**	PTGS2 | RIPK1 | MAP3K7 | TNFAIP3 | IL6 | CREB3 | TRAF2 | NFKBIA |FOS | RPS6KA5 | MAPK3 | MAP2K3 | CCL2 | SOCS3 | JAG1 | CEBPB | JUNB | CREB3L1 | MMP3
**IL-17**	IL-17 | JUN | S100A9 | S100A8 | PTGS2 | CXCL8 | CXCL6 | CXCL1 | CXCL3 | CXCL2 | MAP3K7 | TNFAIP3 | IL6 | TRAF2 | MMP1 | MMP13 | TBK1 |NFKBIA | FOS | MAPK3 | CCL2 | CSF3 | CEBPB | FOSB | MMP3
**NF-kappa B**	CXCL8 | CXCL1 | CXCL3 | CXCL2 | CD14 | RIPK1 | LTB | MAP3K7 | TNFAIP3 | LY96 | TRAF2 | CXCL12 | PLAU | IRAK4 | TNFSF13B | NFKBIA | CCL4 | GADD45B | CCL13 | LBP
**PI3K/Akt**	PKN2 | MCL1 | LAMB3 | PPP2R5A | SOS1 | COL6A3 | FGFR3 | F2R | PDGFRB | TNXB | SGK1| IL6 | COL1A2 | LAMB1 | ANGPT1 | MYC | CREB3 | NTRK2 | TNC | IGF2 | VEGFB | ITGB1 | DDIT4 | NR4A1 | EIF4B | KITLG | COL4A2 | PPP2R3C | THBS1 | FGF7 | MAPK3 | CSF3|COL1A1 | PRKCA | COMP | COL6A2 | IBSP | CREB3L1 | ITGA8 | COL4A4 | THBS4 | LAMC3
**AGE/RAGE**	JUN | AGTR1 | CXCL8 | EGR1 | IL6 | COL1A2 | VEGFB | PLCE1 | COL4A2 | MAPK3 | CCL2 | COL1A1 | PRKCA | BAX | SERPINE1 | COL4A4
**TGFβ**	ID2 | LTBP1 | ACVR2A | SMAD5 | ID4 | MYC | BAMBI | DCN | THBS1 | FBN1 | MAPK3 | SMURF2 | ID1| BMP4 | BMP5
**TLR**	JUN | CXCL8 | CD14 | RIPK1 | MAP3K7 | IL6 | LY96 | IRAK4 | TBK1 | NFKBIA | FOS | MAPK3 | MAP2K3 | CCL3 | CCL4 | LBP
**MAPK**	JUN | ELK4 | MAPKAPK2 | SOS1| CACNA2D3 | FGFR3 | PPP3CA | CD14 | PDGFRB | DUSP1 | HSPA1A | HSPA1B | MAP3K7 | HSPB1 | ANGPT1 | MYC | NTRK2 | TRAF2 | IGF2 | VEGFB | HSPA8 | IRAK4 | NR4A1| KITLG | PPM1A | HSPA2 | FOS | RPS6KA5 | FGF7 | MAPK3 | MAP2K3 | PRKCA | MKNK2 | GADD45B | CACNA1A
**p53**	SFN | RRM2 | ATR | IGFBP3 | CD82 | EI24 | AIFM2 | THBS1 | PMAIP1 | GADD45B | BAX | SERPINE1
**Apoptosis**	JUN | MCL1 | CTSS | TNFSF10 | PTPN13 | RIPK1 | ACTB | CTSB | ENDOG | TRAF2 | CTSD | CTSC | NFKBIA | FOS | MAPK3 | ACTG1 | PMAIP1 | GADD45B | BAX
